# Relationships of Sexual Dysfunction with Depression and Acceptance of Illness in Women and Men with Type 2 Diabetes Mellitus

**DOI:** 10.3390/ijerph14091073

**Published:** 2017-09-16

**Authors:** Ewelina Bąk, Czeslaw Marcisz, Sylwia Krzemińska, Dorota Dobrzyn-Matusiak, Agnieszka Foltyn, Agnieszka Drosdzol-Cop

**Affiliations:** 1 Faculty of Health Sciences, University of Bielsko-Biala, 43-309 Bielsko-Biala, Poland; ewelina.bak76@wp.pl (E.B.); agnieszka_foltyn@wp.pl (A.F.); 2 Department of Gerontology and Geriatric Nursing, School of Health Sciences, Medical University of Silesia, 40-752 Katowice, Poland; klinwewtychy@poczta.onet.pl; 3 Department of Clinical Nursing Faculty of Health Sciences, Medical University, 50-367 Wroclaw, Poland; s.krzeminska@wp.pl; 4 Department of Nursing Propaedeutics, School of Health Sciences, Medical University of Silesia, 40-752 Katowice, Poland; dorotadobrzyn@op.pl; 5 Chair of Woman’s Health, School of Health Sciences in Katowice, Medical University of Silesia, 40-752 Katowice, Poland

**Keywords:** sexual disorders, type 2 diabetes, mental condition of patients, acceptance of illness

## Abstract

An increased prevalence of sexual disorders has been reported in patients with type 2 diabetes. The aim of this study is the assessment of the influence of the psychical condition, the concentration of glycated hemoglobin, the duration of diabetes, the body mass index, the age, and the subjective acceptance of the illness on sexual disorders occurring in women and men with type 2 diabetes. The study enrolled 215 patients (114 women and 101 men) with type 2 diabetes and 183 controls. Sexuality was determined in all of the studied subjects using: the Female Sexual Function Index (FSFI) in women and the International Index of Erectile Function (IIEF) in men. The occurrence of depression symptoms was determined using the Beck Depression Inventory (BDI), whereas the acceptance of the illness in diabetic patients using the Acceptance of Illness Scale (AIS). A sexual dysfunction was found in 68% of the studied diabetic women and 17% of controls. The point values of all the examined FSFI domains were significantly lower in women with diabetes than in controls (*p* < 0.001). Erectile disorders occurred in 82% of the studied men with diabetes and in 41% of the controls (*p* < 0.001). The point values of all the domains of FSFI and IIEF demonstrated a significantly negative correlation with the total BDI score, which was higher in patients with diabetes than in patients without diabetes, and a positive correlation with the total AIS score (*p* < 0.001). The occurrence of sexual dysfunction in patients with diabetes correlated with the age and the duration of diabetes. We conclude that sexual disorders in patients with type 2 diabetes demonstrate the correlation with the occurrence of depression and the acceptance of their illness. Sexual disorders in diabetic patients occur more frequently in older patients and in those with a longer duration of diabetes.

## 1. Introduction

The course of diabetes includes the occurrence of complications and many functional disorders of the organism, i.a. sexual disorders [1,2,3,4,5,6,7], as well a as lowering of mood and depression [8,9].

In women with type 2 diabetes, sexual disorders in the scope of the analyzed domains such as: sexual desire, sexual arousal, orgasm, sexual satisfaction, dyspareunia, and lubrication occurred quite commonly and included from 50% to even 80% of female patients [2,3,6,10,11]. These disorders basically intensified together with age [1,2,6,12,13] and together with the duration of diabetes [1,6,12]. Many authors highlighted that the frequency of occurrence and the intensification of sexual disorders in women with type 2 diabetes was related to the co-occurrence of depressive symptoms [2,10,11,14,15,16]. It has been demonstrated that depression symptoms occurring in women with diabetes caused weaker experiencing of arousal, desire, and orgasm [15,16,17]. On the basis of randomized and multicenter studies Wing et al. [11] demonstrated that the factors which have got an influence on the improvement of the sexual functions in women with type 2 diabetes are the intensification of the lifestyle, especially applying physical activity and the reduction of the increased body mass.

Sexual disorders in men with type 2 diabetes have been described quite broadly, and they basically referred to the erectile dysfunction that occurred among from 35% to even 90% of these patients [4,5,7,18,19,20,21,22]. The intensification of the erectile dysfunction in patients with diabetes was related to their age [1,18,19,20], the duration of the illness [1,7,19], the poorly controlled course of diabetes [7,18,22], depressive symptoms [18,21,23,24], as well as physical activity [18].

Despite of the fact that the reasons for the occurrence of diabetes and its complications have been explored quite thoroughly, the influence of this chronic disease on the occurrence of sexual function disorders in both women and men still has not been fully clarified. The study results presented in the literature which include the assessment of sexual disorders occurring in women and men with type 2 diabetes with taking into consideration the psychological and the somatic factor vary depending on the applied research methods and the sizes of the groups [5]. This premise became the basis for carrying out the present research, which aims at assessing the influence of the psychical condition, the concentration of glycated hemoglobin (HbA1c), the duration of diabetes, the body mass index, the age, and the subjective acceptance of the illness on sexual disorders occurring in women and men with type 2 diabetes.

## 2. Materials and Methods

### 2.1. The Studied Subjects

The study enrolled a population of 215 patients with type 2 diabetes, including 114 women aged from 21 to 65 (mean ± SD = 51.1 ± 9.6) (Group I) and 101 men aged from 33 to 60 (53.3 ± 6.8) (Group II) as well as 183 healthy persons constituting the controls including 94 women aged from 41 to 60 (50.2 ± 5.8) (Group CI) and 89 men aged from 41 to 60 (51.2 ± 5.7) (Group CII). The female patients from Group I were selected out of 151 randomly chosen patients with type 2 diabetes. The male patients from group II were selected out of 145 randomly chosen patients with type 2 diabetes. The subjects for the control groups were selected out of 126 randomly chosen healthy women and 124 randomly chosen healthy men. The reason for reducing the number of patients and the controls qualified for further studies were incompletely filled surveys. The inclusion criterion to the group of studied patients was the occurrence of type 2 diabetes lasting for at least one year and also all of the studied subjects had to have prior sexual initiation. The persons excluded from the study were patients in whom acute inflammation requiring treatment occurred during the last three months, patients taking immunosuppressive drugs, glucocorticoids, anti-inflammatory drugs, as well as patients with diagnosed cancer, endocrine gland diseases, alcoholism, and patients who did not provide consent for performing the study. The treatment of diabetes consisted in adhering to a diet, taking derivatives of sulfonylurea and biguanidines, and in some cases, insulin. All of the ill studied subjects were patients of the Regional Hospital in Bielsko-Biala (the Diabetic Unit), the Diabetic Unit of the Medi-Diab Non-Public Medical Center, and the Diabetic Unit in Katowice. The controls’ examinations were carried out in a Primary Care Outpatient Clinic. The research was carried out in the period from March 2016 untill February 2017. The research was carried out with the consent of the Bioethics Committee of the Beskidzka Regional Chamber of Physicians in Bielsko-Biala; the consent was provided during the meeting held on 11 February 2016 (No. of consent 2016/02/11/1).

### 2.2. Methods

The values of the glucose measures (fasting glycemia, glycemia 2 h after a meal and the concentration of glycated hemoglobin—HbA1c) and the lipid measures (the concentration of LDL-, HDL-, total cholesterol and triglycerides) were determined in all the studied persons. The results of biochemical examinations were obtained on the basis of the medical documentation from the diabetes clinic for patients with diabetes and from the primary care outpatient clinic for the controls. The systolic blood pressure and the diastolic blood pressure were measured.

#### 2.2.1. The Patients’ Demographic and Clinical Data Survey

The questionnaire was accompanied by brief information about the studied person including parameters such as: age, sex, the place of residence, education, marital status, professional activity, body mass, height, used stimulants, comorbidities, the duration of diabetes, the occurrence of diabetes complications, and whether the participant had taken drugs. In case of the studied women, the reproductive history was also assessed.

Before commencing the study, every person was informed about its purpose. The questionnaire was filled in personally and anonymously by the patients and the controls during a routine medical appointment at the diabetes clinic (the group of patients with diabetes), and at the primary care outpatient clinic (the controls). The time needed for filling in the survey was 15–20 min.

#### 2.2.2. Female Sexual Function Index (FSFI)

The FSFI questionnaire developed by Rosen et al. [25] consists of 19 questions allowing for the multidimensional assessment of female sexual functions in relation to the period of the last four weeks. The index has been standardized and adjusted in the Polish language version to differentiating sexual dysfunctions in women aged 18–70 in accordance with the current classifications and recommendations of scientific associations (Polish Society of Sexology, European Society for Sexual Medicine, World Association for Sexual Health) [26]. The questionnaire possesses documented credibility, sensitivity, reliability, internal consistency, as well as stability and repeatability of the results in recognizing disorders of sexual desire, sexual arousal, orgasm, and dyspareunia [25,27]. The questions presented in the questionnaire have been grouped into six domains: sexual desire (two questions), sexual arousal (four questions), lubrication (four questions), orgasm (three questions), sexual satisfaction (three questions) and dyspareunia (three questions). For individual domain scores, the scores of the individual items were added and multiplied by the domain factor. The final results are obtained separately for each of the subscales by summing up the elementary points, which are a part of each of the domains and taking into consideration the designated coefficient; the results are also obtained globally (global assessment).

For the assessment of the particular domains the point score, which may be obtained ranges from 0 to 6.0 for sexual arousal, lubrication, orgasm, and dyspareunia, from 1.2 to 6.0 for the sexual desire function, and from 0.8 to 6.0 for sexual satisfaction. A result below 65% of the maximum score possible to be obtained in each of the domains was considered a sexual disorder of the given function; results below 3.9 points were treated as a sexual dysfunction within the scope of the given domain. In the global assessment, the possible score ranges from 2 to 36 points. A sexual dysfunction is recognized when the total score is equal or lower than 26.5 [27].

The Cronbach α value of FSFI for our results was estimated at the level of 0.96 for type 2 diabetes patients and 0.96 for controls, showing high reliability.

#### 2.2.3. International Index of Erectile Function (IIEF)

The International Index of Erectile Function (IIEF) is a research tool for the investigation of male sexual functions. IIEF is a multidimensional instrument for 5-grade self-assessment of all of the domains of male sexual functions in relation to the period of the last four weeks. This index received standardization for differentiating sexual dysfunctions in men (aged 19–82) in accordance with the international consensus and it is available in 32 language versions, including Polish. It is characterized by high credibility, reliability, sensitivity, and repeatability in the detection of changes confirmed in over 50 clinical trials [28]. The application of the IIEF in the original or the shortened version (IIEF-5) is a recommended standard in the recognizing and assessment of the erectile dysfunction severity grade [28]. The IIEF questionnaire includes 15 items grouped in five collective domains (subscales) describing: I—erection (six questions), II—orgasm (two questions), III—sexual desire (two questions), IV—intercourse satisfaction (three questions) and V—overall satisfaction (two questions). The total scores within domains (I–V) create a positive dependence with correct sexual functioning.

Additional analysis of the erectile subscale facilitates the isolation of four disorder intensification levels (Erectile Dysfunction—ED): erectile function (26–30 points), mild ED (17–25 points), moderate ED (11–16 points), and severe ED (6–10 points).

Clinically significant erectile dysfunctions are diagnosed at values equal to or less than 25 points (cut-off point). The participant rated each item from 1 “very low” to 5 “very high” [28].

The Cronbach α value of IIEF for our results was estimated at the level of 0.96 for type 2 diabetes patients and 0.91 for controls, showing high reliability.

#### 2.2.4. Beck Depression Inventory (BDI)

The Beck Depression Inventory (BDI) is a 21-point screening tool used for assessing the degree of intensity of mood disorder symptoms (depression). The scale consists of 21 questions evaluated from 0 to 3 points. The results obtained in the BDI vary in the range 0–63. A score below 10 points is considered normal. Mild depression is suggested by a score within the range 10–15, moderate depression—16–23, and severe depression—above 24 [29]. The Cronbach α value of BDI for our results was estimated at the level of 0.90 for type 2 diabetes patients and 0.82 for controls, showing a high reliability.

#### 2.2.5. Acceptance of Illness Scale (AIS)

The Acceptance of Illness Scale (AIS) is a questionnaire prepared by Felton et al. and adapted by Juczynski [30]. It includes eight statements, for each of them the examined patient determines his or her current condition in a five-point scale (from 1 to 5). The total score of all of the points is the general measure of the degree of illness acceptance and its scope fits within the range from 8 to 40 points. A low result indicates the lack of acceptance, adaptation to the illness, and a strong feeling of discomfort. A high result, in turn, indicates the acceptance of the illness state, which manifests itself with a lack of negative emotions related to the illness.

### 2.3. Statistical Calculations

The statistical analysis was performed in the Statistica v.10 StatSoft Polska software. The level of statistical significance assumed in all of the calculations was α = 0.05. For quantitative data, the basic statistics were calculated, i.e., the mean value together with the standard deviation. The normality of data distribution was verified using the Shapiro-Wilk test. Due to the fact that the data were not described with normal distributions, the Mann-Whitney U nonparametric test was used for the comparisons of two not connected groups in all of the analyzed cases. Ordinal data were analyzed using the following tests: the Chi-square test, the Yates-corrected Chi-square test, or Fisher's exact test, depending on the observed values and the calculated expected values. This was a way of checking the dependencies between the diabetic group and the control group in ordinal sociodemographic data, in the occurrence of sexual disorders (occurring disorders/lack of disorders) and in the occurrence of depression (occurring depression/lack of depression). Due to the lack of normal distributions, the analysis of variable correlation was performed based on the Spearman rank correlation coefficient. For both the female and the male group correlations were determined between the score obtained for the questionnaire assessing sexuality (FSFI and IIEF), depression problems (BDI), and acceptance of the illness (AIS). The logistic regression model was created in order to verify which of the factors (the patient’s age, BMI, the time (expressed in years) which elapsed from the diagnosing of the disease, the concentration of glycated hemoglobin HbA1c, the occurrence of disease complications, and the mean arterial pressure (MAP) may have a significant influence on the occurrence of disorders in the assessment of sexual functioning. The assessment was performed in both of the analyzed sex groups. The significance of the created model was verified using the Chi-square test. The sufficient goodness of fit of the model was checked using the Hosmer-Lemeshow test and described with the R2 coefficient calculated using the Nagelkerke method.

The reliability of the applied questionnaires was checked by calculating the α-Cronbach coefficient.

## 3. Results

### 3.1. General Characteristics of the Study Groups

Both of the studied groups (Group I and II) were older in comparison to respective control groups CI and CII, and they differed significantly in body mass index, waist circumference, waist to hip ratio, systolic and diastolic blood pressure, glucose fasting, glucose post-meal, total cholesterol, LDL-, HDL-cholesterol, and triglyceride levels, as compared with healthy controls (Table 1). The remaining results assessing the demographic and clinical data have been presented in Table 1.

The analysis of the reproductive history in the group of women with and without diabetes allowed for concluding that the mean age of sexual initiation was 18 ± 2 years in Group I and 17 ± 1 years in the CI Group (the Mann-Whitney U test: *p* = 0.0001). Menarche in Group I: 14 ± 1 years, in the CI Group: 13 ± 1 years; *p* < 0.0001), the duration of the cycle—Group I: 29 ± 2 days, the CI Group: 29 ± 3 days (*p* = 0.2246), the duration of menstrual bleeding—Group I: 5 ± 1 days, the CI Group: 5 ± 2 days (*p* = 0.1254). The number of pregnancies was 2 ± 1 for both groups and the number of childbirths for both groups was also 2 ± 1 (despite of equal mean values, the data distributions differed in a statistically significant way – the results of the Mann-Whitney U test: *p* = 0.0180 for the number of pregnancies and *p* = 0.0092 fort the number of childbirths. The age of sexual initiation in men was 18 ± 2 years in Group II and 18 ± 1 in the IIC Group (*p* = 0.1251).

### 3.2. Assessment of Sexual Function and Depressive Symptoms in Women

Women with type 2 diabetes had significantly lower scores in all six domains (sexual desire, sexual arousal, lubrication, orgasm, sexual satisfaction, and dyspareunia) of the FSFI in comparison to the controls (*p* < 0.0001; Table 2). Seventy-seven (67.5%) women with type 2 diabetes and only 16 (17%) control women had a total FSFI score ≤26.55 (out of the possible total 36 points), and the total FSFI score was lower (worse sexual functioning) in the case of the presence of type 2 diabetes (*p* < 0.0001). The mean BDI score was significantly higher in women with type 2 diabetes than in the control women (*p* < 0.0001). Depressive symptoms were experienced by more women with type 2 diabetes −59.6% than controls −21.3% (*p* < 0.0001) (Table 2).

### 3.3. Assessment of Sexual Function and Depressive Symptoms in Men

Men with type 2 diabetes had significantly lower scores in five domains (erection, orgasm, desire, sexual satisfaction, overall satisfaction) of the IIEF in comparison to the healthy controls (*p* < 0.0001; Table 3). Eighty two (81.2%) men with type 2 diabetes and 41 (46.1%) control men were diagnosed with erectile dysfunctions. The mean BDI score was significantly higher in men with type 2 diabetes than in controls (*p* < 0.0001). Depressive symptoms were experienced by more men with type 2 diabetes −42.6% than controls −4.5% (*p* < 0.0001) (Table 3).

### 3.4. Acceptance of Illness Scale (AIS)

The average value of the AIS in women was 27.7 ± 8.3 points and in men 29.0 ± 8.4 points. The obtained results demonstrate the patients’ moderate level of acceptance of the illness (the AIS scale score range = 8–40 points) and they are similar in both the sexes (*p* = 0.2241).

### 3.5. Sexual Dysfunctions and Diabetic Complications and Glycated Hemoglobin Level

Mean values of total FSFI in diabetic women with (22.66 ± 6.94) and without (24.36 ± 5.90) complications (diabetic foot, neuropathy, nephropathy, retinopathy) were similar (*p* > 0.05) but in men with diabetic complications the score for erection was significantly lower (15.72 ± 7.21) than without diabetic complications (19.63 ± 8.45) (*p* = 0.0203).

Type 2 diabetic women and men with sexual dysfunction had statistically significantly higher levels of HbA1c as compared to women and men without sexual dysfunction (*p* < 0.05).

### 3.6. Sexual Dysfunctions and Demographic Characteristics and Comorbidities

As a result of the statistical analysis, taking into consideration the studied demographic parameters and comorbidities in the group of patients with diabetes it was demonstrated that sexual disorders occurred more frequently only in the group of women who were less educated and those inhabiting rural areas. The frequency of occurrence of sexual disorders in women and men with diabetes was not differentiated depending on the co-existence of comorbidities, such as hypertension, coronary artery disease, and heart failure (Table 4).

### 3.7. Correlation Studies

In all women, the mean total FSFI score and its six domains in diabetics and five domains in controls inversely correlated with the total BDI score (total FSFI: women with type 2 diabetes: r = −0.50, *p* = 0.0001; controls: r = −0.27, *p* < 0.01). In diabetic men, only four out of five domains were significantly inversely correlated with depressive symptoms in the BDI score (erection: men with type 2 diabetes: r = −0.24, *p* < 0.05; controls: r = −0.09, *p* > 0.05) (Table 5).

There was no significant correlation between orgasm and depressive symptoms. In the male control group significant correlation was only demonstrated between overall satisfaction and depressive symptoms (*p* = 0.013) (Table 5).

In both diabetic women and men, FSFI and IIEF total scores positively correlated with the total AIS score (total FSFI: r = 0.44, *p* = 0.0001; erection: r = 0.37, *p* = 0.0001). The patients with better sexual functioning manifested higher adaptation to the illness (Table 5).

The logistic regression analysis of clinically significant FSFI and IIEF values in diabetics facilitated the marking of the odds ratio (OR) for the occurrence of female and male sexual dysfunction depending on the presence of independent variables: age, BMI, duration of diabetes, HbA1c, complications, and mean arterial pressure (Table 6). The occurrence of sexual dysfunction significantly depends in diabetic patients on the age and the duration of diabetes, respectively: in women *p* = 0.0042 and *p* = 0.0012, in men *p* = 0.0178 and *p* = 0.0094.

The likelihood of diagnosing female sexual dysfunction is higher with every passing year of the female patient’s life (odds ratio: 1.11) and with every year elapsing from the moment of diagnosing the illness (odds ratio: 1.23) (Table 6). The performed analysis allowed for concluding that the created model was statistically significant which was proven by the result of the likelihood ratio test (x^2^ = 56.86; *p* < 0.0001). The model’s goodness of fit R^2^ = 0.55 (Nagelkerke). The sufficient goodness of fit was confirmed by the result of the Hosmer-Lemeshow test (*p* = 0.8506).

The likelihood of the occurrence of erectile dysfunction in men with diabetes increases with the male patient’s age (odds ratio: 1.13) and with every year elapsing from the moment of diagnosing the illness (odds ratio: 1.33) (Table 6). It may be concluded that the created model was statistically significant, which was proven by the result of the likelihood ratio test (x^2^ = 34.10; *p* < 0.0001). The model’s goodness of fit R^2^ = 0.48 (Nagelkerke). The sufficient goodness of fit was confirmed by the result of the Hosmer-Lemeshow test (*p* = 0.7847).

The study found no significant influence of BMI, HbA1c, the mean arterial pressure, and the occurrence of complications on the occurrence of sexual disorders in patients with diabetes.

## 4. Discussion

In our own research, we have demonstrated that sexual disorders in patients of both sex with type 2 diabetes occurred significantly more frequently than in persons without diabetes.

In the studied women with diabetes, the total FSFI score was 23.75 and it was by 22% lower than in the women from the control group. In women with type 2 diabetes, the values of this indicator demonstrated in the research of Wing et al. [11] were similar to those obtained by us, whereas in the research of Ogbera et al. [14] they turned out to be lower, which could prove that the sexual disorders observed among those women were even more intensified than in our research. Among all of the six domains determining sexual functions of women with diabetes, we demonstrated the occurrence of the greatest sexual disorders in the scope of desire (70% of the studied women) and arousal (54%), whereas the weakening of the remaining functions (lubrication, orgasm, sexual satisfaction, dyspareunia) occurred in 32.5–35% of women. In the women from the control group, the dysfunction of sexual function did not exceed 10% of the studied persons for any of the domains. The results of this study turned out to be convergent with the data present in the literature, in which it has been indicated that the most frequent sexual dysfunctions occurring in the population of diabetic women were, respectively, the weakening of desire and of arousal [11,15,31].

The likely mechanism of the occurrence of arousal disorders in women with diabetes may be associated with the insufficient vascular flow within the pelvis minoris, as well as with autonomic and peripheral neuropathy [32]. The disorders related to orgasm observed by us occurred significantly more frequently in the group of women with type 2 diabetes, in comparison to the control group, which proved to be compliant with the study results of Olarinoye et al. [12]. Sexual disorders related to dyspareunia occurred in 1/3rd of the women with diabetes examined by us. According to other authors this ailment occurred in women with diabetes slightly less frequently [15] or significantly more frequent [31,33] than in our own research. Dyspareunia during sexual intercourse in women with diabetes may be caused by reduced lubrication within the genital organs [34] and by psychical factors [33]. In our studies, this may be confirmed by the convergent frequency of the occurrence of dyspareunia, reduced lubrication, and lowered sexual satisfaction. A similar convergence of the occurrence of disorders in the scope of two domains (dyspareunia, reduced lubrication within the genital organs) was observed by Nowosielski et al. [15] and by Doruk et al. [31], noting that the latter authors observed these disorders among the majority of the studied women with diabetes. The discrepancies of the obtained study results may have been associated with the selection of the population for the research and with the insufficiently numerous group of the studied women.

In the population of men with type 2 diabetes examined by us the applied research tool was the IIEF questionnaire, which allowed for concluding that erectile dysfunctions occurred in about 80% of the studied patients and these dysfunctions proved to be nearly twice as frequent as in men without diabetes. A similar frequency of erectile dysfunctions among men with type 2 diabetes was demonstrated by El-Sakka and Taveb [7], Dęmbe et al. [35], Sasaki et al. [4] as well as Malavige et al. [19], whereas lower frequency of occurrence of this disorder was observed by Sharifi et al. [20] and De Berardis et al. [21]. The reason for the differentiation in the manifestation of sexual disorders in the scope of erectile dysfunction among men with diabetes could have been factors such as: the cultural aspect, the research methodology, the age of the patients, and the duration of diabetes.

The multifactorial analysis in the logistic regression model allowed us to present the statistically significant risk factors for sexual disorders in the studied men and women with type 2 diabetes. The occurrence of sexual functioning disorders both in men and in women demonstrated a significant relationship with the age of the patients (odds ratio: women 1.11, men 1.13), and the duration of diabetes (odds ratio: women 1.23, men 1.33). This observation corresponds to study results obtained by Olarinoye et al. [12], and Fatemi and Taghavi [13] who described the occurrence of correlations between the age as well as the duration of the illness and sexual disorders in women with type 2 diabetes. Sharifi et al. [20] and Giugliano, et al. [18] demonstrated that the age of men constituted a risk factor for sexual disorders. Ziaei-Rad et al. [1] did not observe a significant influence of age as a risk factor of sexual disorders in men and women with diabetes.

In the present study, it has been found that diabetic women and men with sexual dysfunction had significantly higher levels of HbA1c than those without sexual disorders. However, in correlation studies among the examined patients connections were not demonstrated between the occurrence of sexual disorders and the concentration of HbA1c, so these disorders do not seem to be related to the changes of the concentration of HbA1c. Other studies [18,22] indicate that the factor causing the intensification of erectile dysfunctions in men with type 2 diabetes was worse diabetes control, which was confirmed by an examination of the concentration of HbA1c.

In the own research it has been demonstrated that the incidence of lowered mood in women with type 2 diabetes was nearly three times as high as in the control group (60% vs. 21%). The correlation between the BDI score and the studied domains of sexual disorders occurring in women has been found as highly statistically significant.

It was therefore proven that the higher the degree of depression intensification in ill women the more intensified the disorders of sexual functioning. In the control group, such a dependency was much weaker, and in the case of the orgasm domain it was not observed at all. The research carried out by Bonierbale et al. [36], indicates that in female patients with depression, sexual disorders—especially in terms of desire—occurred significantly more frequently than in women without depression. In the men with type 2 diabetes studied by us, symptoms of depression determined on the basis of the BDI score were observed in 42.6% of patients, while in the control group only in 4.5%. De Berardis et al. [23] demonstrated that the incidence of mood disorders among men with type 2 diabetes depends on psychological factors. Similarly, as in case of the studied women, in men with diabetes we demonstrated the occurrence of significant correlations between the BDI score and sexual disorders with the exception of the orgasm domain. In the control group, such a dependency was observed only within the scope of overall satisfaction. Other authors also demonstrated that in men with type 2 diabetes erectile dysfunctions were related to depressive symptoms [18,21,23,24].

The present studies have also taken into consideration the frequency of occurrence of sexual disorders in patients with type 2 diabetes depending on the studied demographic parameters and the co-existing diseases of the cardiovascular system. It was found that sexual disorders occurred more frequently only among the studied women—in those who were less educated and those inhabiting rural areas. In the study of other authors [13] connections between sexual disorders and the level of education were not observed among women with type 2 diabetes. The frequency of sexual disorders was not differentiated depending on the occurrence of co-existing diseases of the cardiovascular system (hypertension, coronary artery disease, and heart failure). The lack of the dependence between sexual disorders and arterial hypertension in patients with diabetes was also demonstrated by other authors [1]. However, it has to be noted that erectile dysfunction and female sexual dysfunction are common among patients with cardiovascular diseases [37].

In the studies related to the self-acceptance of the illness carried out using the AIS, we demonstrated that the vast majority of the patients studied by us declared the acceptance of their illness. It turned out that the higher the declared level of the patient’s acceptance of his or her illness, the lower the intensity of occurrence of sexual functioning disorders, which has been demonstrated in correlation studies. It should be underlined that studies related to the occurrence of sexual functioning disorders in patients with diabetes in connection with their self-acceptance of the illness have not been carried out until now.

This is one of the first studies in Poland related to both depression, the sexuality of women, and men with type 2 diabetes, as well as the level of acceptance of their illness, which—thanks to the application of multifactorial statistical analysis—allowed for the specifying of risk factors for sexual disorders. However, we are aware of some limitations of this study. Firstly, the cross-sectional and not prospective nature of the study certainly does not allow for the consideration other factors, which in the context of time could modify sexuality. Secondly, as in other self-report inventories (FSFI/IIEF, BDI and AIS), the scoring systems are subjective in nature. Thirdly, in the case of sexual disorders the authors did not consider the lack of satisfaction as a factor discriminating the occurrence of clinically significant sexual disorders, although it seems that introducing such a scale would not affect the study results. The aim of the study was the assessment of the occurrence of disorders of particular sexual functions and not their intensity. Finally, the study and control groups differed significantly with respect to age and education level. The controls were slightly younger and more educated than the study group. These differences might influence the research results, especially sexual function.

## 5. Conclusions

Patients with type 2 diabetes suffer from sexual disorders which occur in 68% of women and 81% of men. The sexual disorders in patients with type 2 diabetes demonstrate a positive correlation with the occurrence of depression, which occurs more frequently in persons with diabetes than in those without it; the mentioned disorders are less intensified in patients who accept their illness. Sexual disorders in patients with type 2 diabetes occur more frequently in older patients, in patients with a longer duration of diabetes.

## Figures and Tables

**Table 1:** Sociodemographic, clinical and biochemical characteristics of the studied patients and controls.

Parameter		Female			Male		
		Diabetes type 2 (Group I) (*n* = 114)	Controls (Group CI) (*n* = 94)	*p*	Diabetes type 2 (Group II) (*n* = 101)	Controls (Group CII) (*n* = 89)	*p*
Age (years)		51.1 ± 9.7	50.2 ± 5.9	0.0169	53.4 ± 6.8	51.3 ± 5.7	0.0021
BMI (kg/m^2^)		28.8 ± 5.4	21.5 ± 1.4	<0.0001	30.2 ± 5.6	23.7 ± 1.2	<0.0001
Waist circumference (cm)		90.0 ± 15.4	68.7 ± 6.7	<0.0001	100.9 ± 16.1	88.9 ± 7.4	<0.0001
Waist to hip ratio		0.87 ± 0.10	0.77 ± 0.05	<0.0001	0.94 ± 0.10	0.86 ± 0.04	<0.0001
Education	Vocational/primary	70	31	<0.0001 ^1^	52	34	0.0028 ^1^
	Preuniversity	34	33		36	25	
	Higher vocational/university	10	30		13	30	
Place of residence	Urban area	10	31	<0.0001 ^1^	41	24	0.0482 ^1^
	Rural area	104	63		60	65	
Marital status	Separated/divorced	5	10	<0.0001 ^3^	16	14	0.0585 ^3^
	Married/in a relationship	109	73		83	66	
	Widow/Widower	0	11		2	9	
Currently working/Not working		113/1	77/17	<0.0001 ^2^	48/53	79/10	<0.0001 ^1^
Smoking: Never/Past/Present		72/21/21	48/20/26	0.1768 ^1^	23/44/34	49/17/23	<0.0001 ^1^
Drinking of alcohol/Not drinking		29/85	50/44	<0.0001 ^1^	48/53	67/22	0.0001 ^1^
Systolic blood pressure (mm Hg)		131.2 ± 14.3	121.5 ± 4.4	<0.0001	134.0 ± 15.5	121.8 ± 3.5	<0.0001
Diastolic blood pressure (mm Hg)		81.5 ± 11.3	66.6 ± 6.6	<0.0001	83.2 ± 13.3	64.4 ± 5.0	<0.0001
Comorbi-dities	Hypertension	62	4		74	0	
	Coronary artery disease	23	0		32	0	
	Heart failure	11	0		22	0	
Drugs	Oral antidiabetic/insulin/antihiper-tensive/statins	114/43/64/43	-		101/57/80/54	-	
Duration of diabetes (years)		8.93 ± 6.55			9.87 ± 7.60		
Complications of diabetes visual disturbances, nephropathy, polineuropathy, diabetic foot		29/8/17/4			31/13/18/4		
Glucose fasting (mg/dL)		126.9 ± 29.9	89.4 ± 8.0	<0.0001	132.5 ± 31.1	90.0 ± 5.1	<0.0001
Glucose post-meal (120 min) (mg/dL)		145.2 ± 31.0	116.9 ± 9.2	<0.0001	155.2 ± 35.2	120.1 ± 5.6	<0.0001
HbA1c (%)		6.47 ± 1.23	-		6.88 ± 1.32	-	
Total cholesterol (mg/dL)		189.7 ± 39.9	113.4 ± 14.2	<0.0001	180.0 ± 33.5	117.5 ± 12.4	<0.0001
LDL-cholesterol (mg/dL)		106.8 ± 33.9	53.9 ± 6.0	<0.0001	95.2 ± 23.1	54.1 ± 4.9	<0.0001
HDL-cholesterol (mg/dL)		54.6 ± 14.9	64.9 ± 5.3	<0.0001	49.8 ± 18.6	60.5 ± 7.3	0.0001
Triglyceride (mg/dL)		145.3 ± 35.8	100.1 ± 12.8	<0.0001	153.5 ± 52.5	98.2 ± 12.9	<0.0001

BMI = body mass index, HbA1c = glycated hemoglobin, LDL = low density lipoprotein, HDL = high density lipoprotein, *p* = statistical significance of differences, ^1^ Chi-square test, ^2^ Yates-corrected Chi-square test, ^3^ Fisher’s exact test, the remaining p values calculated using the Mann-Whitney U test.

**Table 2:** Sexual function and Beck Depression Inventory (BDI) characteristics in diabetic women and controls.

Domains of Sexual Function	Female (Mean ± SD)				Statistical Significance of Difference (*p*)
	Type 2 Diabetic Patients (*n* = 114)		Controls (*n* = 94)		
	Mean ± SD	Sexual Dysfunction *n* (%)	Mean ± SD	Sexual Dysfunction *n* (%)	
Desire	3.31 ± 1.26	80 (70.2)	4.91 ± 0.79	9 (9.6)	<0.0001
Arousal	3.63 ± 1.24	62 (54.4)	5.07 ± 0.69	3 (3.2)	<0.0001
Lubrication	4.08 ± 1.29	38 (33.3)	5.14 ± 0.66	4 (4.3)	<0.0001
Orgasm	4.13 ± 1.22	37 (32.5)	5.21 ± 0.67	2 (2.1)	<0.0001
Sexual satisfaction	4.23 ± 1.18	40 (35.1)	5.09 ± 0.66	5 (5.3)	<0.0001
Dyspareunia	4.38 ± 1.18	37 (32.5)	5.05 ± 0.69	7 (7.5)	<0.0001
Total FSFI	23.75 ± 6.32	77 (67.5)	30.47 ± 3.51	16 (17)	<0.0001
BDI	12.86 ± 8.27		5.32 ± 5.23		<0.0001

FSFI = Female Sexual Function Index, the p values calculated using the Mann-Whitney U test.

**Table 3:** Sexual function and Beck Depression Inventory (BDI) characteristics in diabetic men and controls.

Domains of Sexual Function	Male (Mean ± SD)		Statistical Significance of Difference (*p*)
	Type 2 Diabetic Patients (*n* = 101)	Controls (*n* = 89)	
Erection	17.81 ± 8.10	26.53 ± 2.87	<0.0001
Orgasm	6.48 ± 3.03	8.91 ± 1.08	<0.0001
Desire	6.20 ± 2.65	8.91 ± 1.05	<0.0001
Sexual satisfaction	8.11 ± 3.52	12.88 ± 1.65	<0.0001
Overall satisfaction	6.34 ± 2.40	8.73 ± 1.23	<0.0001
BDI	9.88 ± 9.51	2.62 ± 3.69	<0.0001

The *p* values calculated using the Mann-Whitney U test.

**Table 4:** Demographic characteristics and comorbidities in diabetic patients with and without sexual dysfunction.

Parameter		Diabetic Female (*n* = 114)			Diabetic Male (*n* = 101)		
		With Sexual Dysfunction (*n* = 77)	Without Sexual Dysfunction (*n* = 37)	*p*	With Erectile Dysfunction (*n* = 82)	Without Erectile Dysfunction (*n* = 19)	*p*
Education	Vocational/primary	49	21	0.0099 ^2^	45	7	0.2472 ^2^
	Preuniversity	26	8		29	7	
	Higher vocational/university	2	8		8	5	
Place of residence	Urban area	2	8	0.0026 ^2^	32	9	0.6828 ^2^
	Rural area	75	29		50	10	
Marital status	Separated/divorced	2	3	0.3271 ^3^	14	2	0.8224 ^3^
	Married/in a relationship	75	34		66	17	
	Widow/Widower	0	0		2	0	
Smoking: Never/Past/Present		42/18/17	27/3/4	0.0514 ^2^	19/35/28	4/9/6	0.9910 ^2^
Drinking of alcohol/Not drinking		19/58	10/27	0.7870 ^1^	35/47	13/6	0.0769 ^1^
Comorbidities	Hypertension	46	16	0.2423 ^3^	62	12	0.5236 ^2^
	Coronary artery disease	21	2		30	2	
	Heart failure	9	2		20	2	

*p* = statistical significance of differences, ^1^ Chi-square test, ^2^ Yate’s-corrected Chi-square test, ^3^ Fisher’s exact test.

**Table 5:** The relationship between Female Sexual Function Index (FSFI) or International Index of Erectile (IIEF) and Beck Depression Inventory and Acceptance of Illness Scale.

Domains of Sexual Function		Beck Depression Inventory				Acceptance of Illness Scale	
		Diabetic Females (*n* = 114)		Controls F (*n* = 94)		Diabetic Females (*n* = 114)	
		r	*p*	r	*p*	r	*p*
FSFI	Desire	−0.48	0.0001	−0.30	0.003	0.40	0.0001
	Arousal	−0.50	0.0001	−0.26	0.0112	0.40	0.0001
	Lubrication	−0.45	0.0001	−0.25	0.0142	0.38	0.0001
	Orgasm	−0.42	0.0001	−0.14	0.1641	0.37	0.0001
	Sexual satisfaction	−0.37	0.0001	−0.23	0.0268	0.36	0.0001
	Pain or Dyspareunia	−0.33	0.0003	−0.27	0.0074	0.35	0.0001
	Total FSFI	−0.50	0.0001	−0.27	0.0084	0.44	0.0001
		**Diabetic Males** **(*n* = 101)**		**Controls M** **(*n* = 89)**		**Diabetic Males** **(*n* = 101)**	
IIEF	Erection	−0.24	0.0168	−0.09	0.3982	0.37	0.0001
	Orgasm	−0.18	0.0772	−0.16	0.1224	0.31	0.0014
	Desire	−0.23	0.0187	−0.15	0.1524	0.33	0.0006
	Sexual satisfaction	−0.24	0.0179	−0.05	0.6492	0.30	0.0022
	Overall satisfaction	−0.28	0.0044	−0.26	0.013	0.36	0.0002

r = Spearman rank correlation coefficient, *p* = statistical significance of difference.

**Table 6:** Results from the logistic regression analysis of sexual dysfunction in diabetics depending on the presence of independent variables.

		Rating	Standard Error	Wald x^2^	*p*	Odds Ratio (OR)	95% CI for OR
FSFI	Intercept term	−12.43	4.34	8.21	0.0042	0.4 × 10^−5^	1.60 × 10^−9^−0.01
	Age	0.11	0.04	8.19	0.0042	1.11	1.03–1.20
	BMI	0.12	0.06	3.81	0.0509	1.12	1.00–1.26
	Duration of diabetes	0.21	0.07	10.44	0.0012	1.23	1.08–1.40
	HbA1c	0.32	0.30	1.19	0.2748	1.38	0.77–2.49
	Diabetic complications	−0.24	0.58	0.16	0.6854	0.79	0.25–2.51
	MAP	0.01	0.03	0.10	0.7542	1.01	0.95–1.08
IIEF	Intercept term	−1.83	4.32	0.18	0.6715	0.16	0.33 × 10^−4^–7.79 × 10^2^
	Age	0.12	0.05	5.61	0.0178	1.13	1.02–1.24
	BMI	−0.03	0.07	0.15	0.7026	0.97	0.85–1.11
	Duration of diabetes	0.28	0.11	6.75	0.0094	1.33	1.07–1.65
	HbA1c	0.34	0.33	1.10	0.2946	1.41	0.74–2.70
	Diabetic complications	1.21	0.80	2.30	0.1293	3.37	0.69–16.53
	MAP	−0.06	0.04	3.17	0.0752	0.94	0.87–1.01

*p* = statistical significance of difference, BMI = body mass index, HbA1c = glycated hemoglobin, MAP = mean arterial pressure, 95% CI = confidence interval.
